# Short Communication on Proposed Treatment Directions in Bipolar Disorder: A Psychotherapy Perspective

**DOI:** 10.3390/jcm14061857

**Published:** 2025-03-10

**Authors:** Jelena Milic, Iva Zrnic, Milica Vucurovic, Edita Grego, Sanja Djurdjevic, Rosa Sapic

**Affiliations:** 1 Institute of Public Health of Serbia “Dr Milan Jovanović Batut”, 112113 Belgrade, Serbia; 2 European Faculty “Kallos”, 11000 Belgrade, Serbia; 3 Regional Medical Chamber of Belgrade, 11111 Belgrade, Serbia; 4 Academy for Human Development, 11111 Belgrade, Serbia; 5 Faculty of Health Studies, University of Bjeljina, Bjeljina 76300, Republika Srpska, Bosnia and Herzegovina

**Keywords:** bipolar disorder, patient-centered care, psychoeducation, Cognitive Behavioral Therapy, mood stabilizers, self-management strategies

## Abstract

**Background/Objectives**: Bipolar disorder (BD) is a chronic, severe mental health condition characterized by episodes of mood instability, including manic and depressive episodes. While pharmacological interventions remain foundational in BD treatment, psychotherapy offers significant benefits by addressing the psychological and behavioral components that contribute to mood episodes and overall functioning. The primary objective of this short communication is to propose new directions in psychotherapy for treating bipolar disorder, focusing on integrative models that combine evidence-based therapies such as Cognitive Behavioral Therapy (CBT), Interpersonal and Social Rhythm Therapy (IPSRT), Family-Focused Therapy (FFT), and mindfulness-based approaches. By integrating these therapies, clinicians can target both cognitive distortions and emotional dysregulation while simultaneously stabilizing sleep–wake cycles and improving interpersonal functioning. The secondary objective emphasizes the importance of better understanding and psychoeducation in family therapy, which can promote a better understanding of BD among family members and ensure more effective management of the disorder in daily life. **Methods**: We explore the potential of Cognitive Behavioral Therapy (CBT), Interpersonal and Social Rhythm Therapy (IPSRT), Family-Focused Therapy (FFT), and mindfulness-based interventions in enhancing symptom management and preventing relapse. **Results**: We identified psychoeducation and family therapy as critical components in supporting patients and improving treatment adherence. These therapeutic interventions play a pivotal role in enhancing patient engagement, improving coping strategies, and facilitating better overall treatment outcomes. **Conclusions**: We propose a multidisciplinary approach, integrating psychotherapy with pharmacotherapy, to optimize long-term outcomes and improve the overall quality of life for individuals with bipolar disorder.

## 1. Introduction

### 1.1. Overview of Bipolar Disorder and Its Management

Bipolar disorder (BD) is a complex mood disorder that presents significant challenges for both patients and clinicians. BD is characterized by extreme mood swings, including manic, hypomanic, and depressive episodes. These mood changes can have a profound impact on an individual’s ability to function socially, occupationally, and personally. BD affects approximately 1–2% of the global population, with a significant burden on individuals, families, and healthcare systems [1]. The disorder is typically managed with pharmacotherapy, including mood stabilizers and antipsychotic medications, which aim to control the acute phases of the disorder. However, pharmacological treatments alone do not address the psychological, social, and behavioral factors that contribute to the recurrent nature of BD. Furthermore, long-term management remains a challenge, as BD often requires continuous treatment across the patient’s lifespan. The recurrence of episodes and challenges in achieving stable remission make BD particularly difficult to treat [2,3].

### 1.2. The Role of Psychotherapy in Bipolar Disorder Treatment

While pharmacotherapy plays a critical role in managing BD, it often fails to address the underlying psychological and emotional aspects that perpetuate the disorder. Published evidence suggests that medication alone is insufficient in reducing the frequency of relapse or improving overall quality of life in BD patients [4]. As a result, psychotherapy has become an increasingly important adjunct to pharmacological treatment. Psychotherapy offers a multifaceted approach to BD management, focusing on helping patients manage mood fluctuations, cope with stress, and address cognitive distortions that can worsen symptoms. Recent studies have shown that combining psychotherapy with pharmacological treatments significantly enhances patient outcomes, particularly in terms of relapse prevention, mood stabilization, and overall well-being [5]. By targeting both the cognitive and emotional dimensions of BD, psychotherapeutic interventions have the potential to transform the course of the illness.

### 1.3. Understanding the Need for Psychotherapeutic Interventions

Bipolar disorder’s complex nature demands a treatment approach that targets not only the biological components but also the emotional, social, and cognitive factors that contribute to the disorder’s recurrence. As pharmacological therapies often fail to address these dimensions comprehensively, the inclusion of psychotherapies is pivotal in achieving long-term stability and quality of life for BD patients. Psychological therapies are effective in addressing the cognitive and behavioral changes that occur during manic, hypomanic, and depressive episodes, and they help patients develop coping mechanisms that are crucial for managing the disorder. Evidence suggests that psychotherapy not only improves mood regulation but also reduces the likelihood of recurrence by addressing the psychosocial factors that trigger mood episodes, such as stress and interpersonal conflicts [6].

### 1.4. Advances in Psychotherapeutic Approaches for Bipolar Disorder

Over the past several decades, multiple psychotherapeutic modalities have been developed and studied in the context of BD treatment. The most widely researched therapies include Cognitive Behavioral Therapy (CBT), Interpersonal and Social Rhythm Therapy (IPSRT), Family-Focused Therapy (FFT), and mindfulness-based interventions. These therapies have gained traction because of their demonstrated effectiveness in reducing symptoms and improving long-term outcomes for BD patients. CBT focuses on altering maladaptive thought patterns that contribute to mood instability, while IPSRT aims to stabilize sleep–wake cycles and social rhythms, which have been shown to directly influence mood regulation [7]. Family-based therapies, like FFT, emphasize educating family members and improving communication within the household to support the patient’s long-term management of the disorder [8]. Mindfulness-based interventions, such as Mindfulness-Based Cognitive Therapy (MBCT), help patients become more aware of their emotional experiences and develop techniques to prevent depressive relapses [9].

### 1.5. What Psychotherapies Are Found to Be Effective in Bipolar Disorder Patients

BD is a complex and multifactorial condition that requires a multifaceted treatment approach. Psychotherapy, when combined with pharmacological interventions, has proven to be highly effective in managing BD, especially in preventing relapse and improving long-term outcomes. In our manuscript, we introduce four specific psychotherapies: Cognitive Behavioral Therapy (CBT), Interpersonal and Social Rhythm Therapy (IPSRT), Family-Focused Therapy (FFT), and mindfulness-based approaches. Each of these therapies has a unique and evidence-based role in treating BD and addressing its various dimensions, including mood regulation, social functioning, and emotional regulation.

### 1.6. Comparative Studies and Selection of Therapies

The selection of CBT, IPSRT, FFT, and mindfulness-based approaches in our manuscript was based on their strong empirical support in the management of BD. Numerous studies have demonstrated that these therapies not only alleviate symptoms but also improve patients’ overall functioning and quality of life. Systematic reviews have shown that psychoeducation, CBT, FFT, IPSRT, and peer support programs are highly efficient in the treatment of BD [4,5]. Additionally, emerging therapies such as functional remediation, mindfulness-based cognitive therapy, illness management and recovery, and technology-assisted strategies show promise [5]. The research consistently demonstrates the benefits of combining these psychotherapies with pharmacotherapy, as compared to using medication alone. Adjunctive evidence-based practices accelerate remission, prolong the time to recurrence, and enhance functional outcomes [4,5]. These therapies are vital in helping individuals build the necessary skills to manage the enduring psychosocial, neurocognitive, vocational, and interpersonal challenges that come with bipolar disorder. By integrating these therapies, clinicians can target both cognitive distortions and emotional dysregulation, while simultaneously stabilizing sleep–wake cycles and improving interpersonal functioning [6,7,8,9]. Further, psychoeducation and family therapy can promote better understanding of BD among family members and ensure more effective management of the disorder in daily life [10]. In line with this, we advocate for a multidisciplinary treatment framework that aligns pharmacological and psychotherapeutic interventions to provide holistic, patient-centered care. In Table A1, we present an overview of the effects of psychotherapy on BD symptoms and cognitive, emotional, and behavioral pathways.

### 1.7. Clinical Implications of Psychotherapy in Bipolar Disorder Treatment

#### 1.7.1. Enhancing Long-Term Stability and Preventing Relapse

In clinical practice, the management of bipolar disorder (BD) involves a careful balance of pharmacotherapy and psychotherapy. While pharmacological treatments such as mood stabilizers and antipsychotics remain the cornerstone of BD management, psychotherapy has increasingly been recognized as a critical adjunct for achieving long-term stability and improving patients’ overall quality of life. One of the primary clinical benefits of psychotherapy is its potential to reduce relapse rates. BD is known for its recurrent nature, with patients experiencing multiple mood episodes throughout their lifetime. By combining psychotherapy with pharmacological interventions, clinicians are better equipped to prevent these relapses and ensure long-term stability [1].

#### 1.7.2. Subtypes of Bipolar Disorder: Type 1 and Type 2

BD is characterized by extreme mood swings, including manic, hypomanic, and depressive episodes [1]. Further, BD is classified into two major subtypes: Bipolar I Disorder (BD-I) and Bipolar II Disorder (BD-II). These subtypes are distinguished by the severity and duration of manic episodes. BD-I is characterized by the occurrence of at least one manic episode, which can be severe and may require hospitalization. Patients with BD-I also experience depressive episodes, often leading to significant impairment in daily functioning. In contrast, BD-II is marked by hypomanic episodes, which are less severe than full-blown mania, but patients still experience depressive episodes. BD-II often presents a more chronic pattern of mood fluctuations, with depressive episodes being more frequent and prolonged. Understanding these subtypes is critical for determining the most effective therapeutic approach for each individual. BD-I may benefit more from interventions aimed at managing manic episodes, while BD-II often requires a stronger focus on preventing depressive episodes, which are more prevalent and disabling in this subtype. Both types, however, benefit from a combination of pharmacotherapy and psychotherapy to address the full spectrum of symptoms and enhance long-term outcomes.

#### 1.7.3. Addressing Medication Adherence and Reducing Psychosocial Stress

Another critical clinical implication is the improvement in medication adherence. BD patients often experience periods of non-compliance with prescribed medications, either due to side effects or a lack of insight into the disorder. Psychotherapy can play a significant role in addressing these challenges by providing patients with education, motivation, and the necessary tools to manage their illness more effectively. Additionally, psychotherapy addresses the psychosocial stressors that often trigger mood episodes, such as relationship difficulties, work stress, or sleep disturbances. As mood episodes are frequently exacerbated by these external factors, addressing them through therapeutic interventions is essential for long-term disease management.

#### 1.7.4. Improving Quality of Life and Social Functioning

Furthermore, psychotherapy has shown promise in improving the overall quality of life for individuals with BD [6]. Through approaches like CBT and IPSRT, patients can develop better coping mechanisms, regulate their emotions more effectively, and improve interpersonal relationships. These improvements often lead to better occupational functioning, greater social integration, and a reduction in the stigma that patients may face. The long-term clinical benefits are particularly evident when psychotherapy is incorporated into the treatment plan early in the course of BD, reducing the long-term burden of the disorder and enhancing overall functioning [6].

#### 1.7.5. Coordinated and Patient-Centered Care

In a clinical setting, the integration of psychotherapy into BD treatment plans supports a comprehensive treatment framework that involves collaboration between psychiatrists, psychologists, therapists, and the patient’s family, ultimately contributing to a more coordinated and effective treatment plan. By addressing the full spectrum of the disorder’s impact—biological, psychological, and social—psychotherapy enhances the holistic care approach needed to manage BD effectively over the long term [1].

### 1.8. Objectives

The primary objective of this short communication is to propose new directions in psychotherapy for treating bipolar disorder, focusing on integrative models that combine evidence-based therapies such as CBT, IPSRT, FFT, and mindfulness-based approaches. By integrating these therapies, clinicians can target both cognitive distortions and emotional dysregulation while simultaneously stabilizing sleep–wake cycles and improving interpersonal functioning. The secondary objective emphasizes the importance of better understanding and psychoeducation in family therapy, which can promote a better understanding of BD among family members and ensure more effective management of the disorder in daily life.

## 2. Findings and Discussion

### 2.1. Introduction to Psychotherapy and Its Role in BD Treatment

The treatment of BD has predominantly been medication-focused. However, psychotherapeutic interventions have gained recognition for their ability to support long-term management and prevent relapse. Psychotherapies, when integrated with pharmacological treatments, offer a comprehensive approach to addressing the multiple facets of BD. The following sections delve into each specific therapy and its proven benefits.

### 2.2. Cognitive Behavioral Therapy (CBT)

CBT is one of the most widely studied psychotherapies for BD, with a robust body of research supporting its efficacy. It primarily targets the cognitive and emotional components of BD, helping individuals identify and change negative thought patterns that can trigger mood episodes. CBT is particularly effective in treating depressive episodes in BD, as it helps individuals challenge maladaptive thinking and develop healthier coping strategies. The biological aspects (i.e., the “bio” component of the bio-psycho-social model) of BD are central to understanding its causes and treatment. However, managing BD with medication alone is challenging, as episode intensity, duration, and type might require different treatments. Considering both the high prevalence of BD and the limited effectiveness of pharmacological treatments, it is clear that psychosocial interventions play a crucial role in its management [11,12]. Furthermore, issues like treatment non-compliance also necessitate psychosocial strategies. CBT, which is often inadequate in its empirical foundation and adaptability, stands out as an evidence-based therapy. It is recommended as an adjunctive treatment throughout most stages of the disorder, excluding acute mania [12]. CBT can reduce the frequency and severity of depressive episodes, as well as improve overall mood stability.

### 2.3. Interpersonal and Social Rhythm Therapy (IPSRT)

IPSRT is based on the idea that stabilizing daily routines, especially sleep–wake cycles, can play a crucial role in managing mood episodes in BD. Research has shown that disruptions in sleep and social rhythms can trigger mood swings in BD patients, making this therapy particularly effective [7,13]. IPSRT focuses on stabilizing these rhythms, which in turn reduces the frequency of mood episodes. Studies, such as those by Frank et al., have shown that IPSRT is particularly beneficial in patients with BD Type I (BD-1) and Type II (BD-2), helping them regulate their daily routines and improve interpersonal relationships, which are often disrupted by the disorder [14].

Moreover, IPSRT places a strong emphasis on addressing interpersonal stressors, which often contribute to mood disturbances. By improving communication skills and fostering stable relationships, patients can better manage social challenges that may exacerbate BD symptoms. Furthermore, maintaining a regular routine reduces the risk of mood destabilization caused by external triggers, such as stressful life events. Studies also suggest that IPSRT can be effective in reducing depressive symptoms and preventing recurrence of mood episodes by helping individuals with BD develop more structured and predictable daily habits, leading to better long-term management of the disorder.

### 2.4. Family-Focused Therapy (FFT)

In the context of bipolar disorder, FFT provides psychoeducation to families about the condition, enabling them to recognize early signs of mood episodes and respond appropriately. It emphasizes enhancing family dynamics, improving communication, and fostering problem-solving skills to mitigate the impact of bipolar disorder on both individuals and their families. By incorporating family involvement, FFT addresses relational stressors that can exacerbate symptoms, thereby creating a supportive environment conducive to stability and recovery. This highlights the importance of family-centered approaches in managing bipolar disorder effectively [8]. Family dynamics play a significant role in the course of BD. FFT focuses on educating family members about the disorder, helping them recognize early signs of mood episodes and providing them with the tools to support the patient effectively. FFT also emphasizes improving communication and problem-solving skills within the family, which is crucial for reducing relational stress, which can exacerbate BD symptoms. Research has shown that FFT can reduce relapse rates and improve overall functioning in patients with BD. For example, studies by Miklowitz et al. have demonstrated that FFT significantly reduces the likelihood of relapse in patients with BD and improves the quality of family relationships, which is essential for long-term stability [8].

### 2.5. Mindfulness-Based Interventions, Including Mindfulness-Based Cognitive Therapy (MBCT)

MBCT and other mindfulness-based interventions have gained attention for their ability to help BD patients develop greater awareness of their thoughts and emotions. By fostering a nonjudgmental awareness of internal experiences, mindfulness-based therapies help patients detach from ruminative thinking patterns that can lead to depressive episodes. MBCT is particularly effective in preventing depressive relapse, especially in patients with BD-2, where depressive episodes are more prevalent [15,16,17]. MBCT and other mindfulness-based interventions have garnered attention for their ability to help individuals with BD develop a deeper awareness of their thoughts and emotions. By cultivating nonjudgmental awareness of internal experiences, these therapies enable patients to detach from ruminative thinking patterns that often contribute to depressive episodes. MBCT has specifically been found to reduce relapse rates in patients with recurrent depressive episodes, which are common in BD [17]. Research demonstrates MBCT’s efficacy in preventing depressive relapse, particularly in patients with BD-2, where depressive episodes are more frequent [17]. Further supporting these findings, a study revealed that following MBCT, patients showed significant improvements in executive functioning, memory, and task initiation and completion, as measured by the Behavior Rating Inventory of Executive Function (BRIEF) and the Frontal Systems Behavior Scale (FrSBe) [18]. These cognitive improvements were linked to increased mindfulness—defined as nonjudgmental observation and awareness of thoughts, feelings, and sensations. Interestingly, these cognitive changes were not directly associated with reductions in depressive symptoms [18]. While the benefits appeared to decrease after the treatment ended, improvements in executive functioning persisted for up to three months. These results offer preliminary evidence that MBCT could serve as a valuable adjunct to medication, particularly for enhancing cognitive functioning in BD patients [18].

### 2.6. Psychoeducation: A Pivotal Role in the Prevention of Relapses and Treatment of BD

In addition to these individual therapies, psychoeducation plays a pivotal role in BD treatment. Educating patients and their families about the nature of BD, recognizing early signs of relapse, and understanding medication adherence are essential for long-term management. Psychoeducation aims to provide individuals and their support system with the knowledge necessary to navigate the complexities of the disorder and maintain better overall functioning.

Psychoeducation reduces stigma, empowers patients, and promotes healthier coping strategies, thus improving outcomes. Patients who are well informed about their condition are better equipped to identify early warning signs of mood swings and engage in timely interventions. Understanding the illness empowers them to make informed decisions about treatment, compliance with medications, and lifestyle adjustments that can positively influence their prognosis. Moreover, psychoeducation can significantly reduce feelings of isolation and confusion, making it easier for patients to cope with the disorder’s impact on their personal and social lives [19].

Family therapy also enhances communication and problem-solving skills within the family unit, providing a supportive environment for the patient [20]. A well-educated family can better respond to crises, reduce misunderstandings, and contribute to more effective management of the disorder. By promoting understanding, psychoeducation helps to prevent relapses by fostering adherence to treatment regimens, while also supporting healthier coping mechanisms that help manage stress and interpersonal challenges associated with BD. In fact, psychoeducation has been shown to reduce relapse rates, particularly when patients are actively engaged in treatment and their family members are involved in the process [19]. We provide Table A2 for an overview of early signs of relapse in BD, with corresponding potential interventions for family members/caregivers, therapists, and the patient themselves.

### 2.7. Psychotherapeutic Approaches in Preventing Relapse in BD

Several psychotherapeutic approaches have shown significant promise in preventing relapse in BD [5,21,22], though more high-quality meta-analyses or randomized controlled trials (RCTs) could further support their long-term effectiveness. Psychoeducation has consistently been demonstrated to help individuals the most with BD by increasing their understanding of the disorder, improving medication adherence, and reducing relapse rates. The results of a systematic review identified the effectiveness of psychoeducation in preventing relapse of mood episodes, but primarily in a selected subgroup of patients at an early stage of the disorder who have achieved good, if not complete, remission from the acute episode [22]. Another systematic review found that psychoeducation reduced relapse rates, improved long-term treatment adherence, and improved the knowledge of the illness for patients and caregivers, resulting in improved social functioning [23]. CBT and IPSRT may offer some benefits during the acute phase, though additional data are needed to confirm their efficacy in relapse prevention [11,22]. Mindfulness-based interventions have been shown to reduce anxiety, but their effectiveness in preventing mood episodes remains limited [22]. On the other hand, interventions aimed at improving neurocognitive function appear to be largely ineffective in preventing relapse [22]. Family-focused interventions seem to provide benefits mainly for caregivers, but it remains uncertain whether they have a direct impact on patient outcomes [22].

Technology-assisted strategies, such as digital CBT or apps for mood tracking, offer patients additional tools to manage symptoms and stay connected with their support system. While these approaches have shown encouraging results, more systematic reviews or meta-analyses are needed to solidify their long-term efficacy in relapse prevention.

### 2.8. Revisiting Family-Focused Therapy and Psychoeducation: Core Pillars for Preventing Relapse in Bipolar Disorder

In our exploration of effective therapies for managing BD, it is important to re-emphasize the vital roles that FFT and psychoeducation play in preventing relapse and improving overall patient outcomes. Although previous sections of this manuscript have already highlighted the relevance of both approaches, the significance of returning to these therapies in our discussion lies in their enduring effectiveness and complementary roles in the comprehensive treatment of BD. While individual therapies have gained traction in the field, these two family-centered strategies continue to offer unique benefits that contribute to long-term stability and recovery for individuals with BD.

FFT’s emphasis on engaging family members as active participants in the treatment process is critical to reducing relapse rates in BD. The treatment consists of conjoint sessions of psychoeducation regarding bipolar illness, communication enhancement training, and problem-solving skills training [8]. The active involvement of families in therapy not only helps patients recognize early warning signs of mood shifts but also builds a supportive network capable of responding effectively to those changes. By fostering healthier family dynamics, FFT helps mitigate relational stressors—often a significant trigger for mood episodes in BD. In addition to providing psychoeducation to families, FFT promotes open communication and the development of problem-solving strategies, which are essential for maintaining a stable and supportive home environment [8]. Moreover, family members are often the first to notice early signs of relapse, and FFT equips them with the skills to intervene early, thus preventing the escalation of symptoms and reducing the frequency of hospitalizations.

Meanwhile, psychoeducation extends beyond the confines of the therapy room to address key aspects of BD management. Educating patients and their families about the nature of the disorder, recognizing the early signs of relapse, and fostering understanding around medication adherence are all crucial components of a comprehensive treatment plan. Psychoeducation also works to reduce the stigma associated with BD, empowering patients and their families to take an active role in their own care. A recent systematic review found that psychoeducation for both patients and their families was associated with fewer new mood episodes and a reduction in the number and duration of hospitalizations [10]. Additionally, psychoeducational interventions with patients were linked to improved adherence to medication. These interventions did not appear to significantly impact the severity of manic or depressive symptoms, nor did they affect the patient’s overall quality of life or functionality [10].

By increasing awareness, patients are better equipped to manage their symptoms, make informed decisions regarding their treatment, and maintain a healthy lifestyle that supports long-term stability. Additionally, psychoeducation encourages patients to develop coping strategies that promote resilience, which is particularly important when managing the cyclical nature of BD.

The value of integrating both FFT and psychoeducation into the treatment plan lies in their synergistic effect. While FFT creates a foundation of support through improved family dynamics, psychoeducation complements this by empowering both the patient and family to manage BD more effectively. As research consistently shows, these approaches are linked to better overall outcomes, such as fewer hospitalizations, reduced relapse rates, and improved quality of life. A previous study found that adolescent bipolar patients benefit the most when family-associated stress is elevated or when persistent hypomanic or manic symptoms are the primary targets of treatment but family members are attending FFT and know how to handle these situations [24]. By educating and involving the family in the therapeutic process, FFT and psychoeducation address both the psychological and relational aspects of BD, leading to better long-term outcomes for patients.

In conclusion, the decision to revisit and emphasize FFT and psychoeducation in the context of BD treatment is not only relevant but necessary. These therapies provide invaluable tools that facilitate early intervention, improve patient and family understanding of the disorder, and offer an ongoing support system that is crucial in managing BD. Their continued importance in the clinical management of BD highlights the need for a comprehensive, family-centered approach that integrates both psychoeducation and therapy to reduce relapse rates, enhance medication adherence, and ultimately improve long-term outcomes for individuals with bipolar disorder. By ensuring that both patients and their families are equipped with the knowledge and skills to navigate the complexities of BD, we can foster a more effective and sustainable model of care.

### 2.9. Understanding Bipolar Disorder Subtypes and Their Role in Tailoring Treatment Approaches

The treatment of BD has traditionally been medication-focused, but psychotherapeutic interventions have gained recognition for their ability to support long-term management and prevent relapse.

To compare the characteristics of BD patients diagnosed as DSM-5 types I vs. II, BD is classified into two subtypes: Type I and Type II, each with distinct characteristics that influence treatment strategies. BD-1 is defined by the occurrence of at least one manic episode, often accompanied by depressive episodes, with manic episodes typically being severe enough to cause significant functional impairment, hospitalization, or psychosis. In contrast, BD-2 is characterized by recurrent depressive episodes and hypomanic episodes, which are less severe than full-blown mania and do not cause significant impairment or psychosis. Research has shown that individuals with BD-2 tend to experience longer depressive episodes and less frequent or less severe manic episodes compared to those with BD-1, with BD-2 patients also presenting with higher socioeconomic status, more education, and fewer hospitalizations [25]. Additionally, BD-2 is associated with higher long-term morbidity, including increased depressive symptoms, and higher suicide risk, despite the less frequent occurrence of manic or psychotic episodes [25]. These patients also show a preference for antidepressant and benzodiazepine treatment over lithium or antipsychotics [25]. These differences are critical when tailoring treatment approaches, as BD-1 requires more intense management of manic episodes, while BD-2 treatment should focus on managing prolonged depressive states and preventing relapse, often with a greater emphasis on antidepressants and anxiolytics.

However, the therapeutic outcomes of treatment interventions can vary significantly between BD-1 and BD-2, and understanding these differences is critical to tailoring treatments for individual patients.

CBT, known to be highly effective in managing BD-1, helps by identifying and reframing negative thought patterns present in depressive symptoms, and aids preventing manic episodes, especially important in BD-1. Studies have shown that CBT can assist patients with either subtype in increasing medication adherence and effectively managing mood triggers, though BD-1 patients may require additional attention to manic symptomatology during treatment [11,12].

IPSRT has shown promise in stabilizing daily routines and sleep–wake cycles, which play a critical role in the onset of mood episodes in BD. IPSRT has demonstrated effectiveness in both BD-1 and BD-2; however, it has been particularly beneficial for BD-1 patients, who often experience more pronounced mood swings, especially manic episodes. For BD-2, where depressive episodes tend to be more frequent, stabilizing daily routines through IPSRT can help prevent the onset of depressive mood episodes triggered by social or environmental disruptions [7,13]. In both subtypes, the stabilization of sleep patterns and daily rhythms can significantly reduce the frequency and intensity of mood episodes.

FFT plays a critical role in managing the relational stressors that often exacerbate BD symptoms. By educating family members about the early signs of mood episodes and providing strategies for effective communication, FFT supports patients in both subtypes by promoting a stable and supportive environment. For BD-1 patients, who may experience more severe manic episodes, family intervention is vital in recognizing early signs of mania, while for BD-2 patients, the focus is often on managing depressive episodes and preventing relapse [8].

CBT, widely recommended for BD-2, effectively manages depressive episodes by reframing negative thought patterns and preventing relapses. Research suggests that CBT helps individuals with BD-2.

Mindfulness-based interventions, such as MBCT, have also shown promise in reducing relapse rates in BD. For BD-2 patients, MBCT is particularly beneficial in managing depressive relapse, as it helps them detach from ruminative thinking patterns associated with depression. In BD-1, MBCT’s ability to improve emotional regulation and reduce reactivity to stressors can help prevent manic episodes, contributing to better overall mood stabilization [15,16]. Mindfulness techniques, by fostering awareness of emotions and reducing impulsivity, serve as an effective intervention in both subtypes, though their impact may be more significant in preventing depressive relapse for those with BD-2.

Finally, psychoeducation plays a pivotal role in BD treatment by helping both patients and their families understand the nature of the disorder, recognize early signs of relapse, and adhere to medication regimens. Psychoeducation is particularly crucial in BD-1, where episodes of mania can be more disruptive, and in BD-2, where recurrent depressive episodes can often lead to long-term functional impairment. By empowering patients and families to recognize symptoms and respond early, psychoeducation serves as a foundational component in the long-term management of both subtypes.

We have summarized early signs of relapse in BD and corresponding interventions for families and caregivers in Table A2, which provides practical strategies for managing symptoms across both subtypes.

### 2.10. Exploring Potential Problems in the Integration of Psychotherapy and Pharmacotherapy for Bipolar Disorder Treatment

The integration of psychotherapy and pharmacotherapy in the treatment of BD is crucial for addressing the complex nature of the condition. However, multiple obstacles exist that hinder the seamless combination of these approaches in clinical practice. One of the most pressing challenges is patient adherence to both pharmacological and psychotherapeutic interventions. Studies show that BD patients often struggle with sticking to their medication regimens, particularly when they feel stable or well, and may also exhibit reluctance toward psychotherapy. About half of the patients diagnosed with BD become non-adherent during long-term treatment [26]. The stigma surrounding mental health treatment is another significant barrier, with many patients feeling ashamed or resistant to engaging in psychotherapy, even when it is a recommended part of their treatment plan. Stigma leads to delayed treatment, increased morbidity, and diminished quality of life for those with poor mental health [27]. This resistance complicates the integration of both treatment modalities, as adherence to one may not necessarily correlate with adherence to the other.

Another key obstacle is the fragmentation of care in clinical settings. Psychiatrists, who primarily oversee pharmacotherapy, often do not coordinate effectively with psychotherapists who provide CBT or FFT. This lack of collaboration can lead to inconsistent treatment plans and delays in adjusting therapy when necessary, thereby diminishing the effectiveness of the integrated approach. However, integrated psychiatric care—where a psychiatrist handles both the medication and psychotherapy—can, in the long run, reduce total costs and lessen patient suffering when compared to the more expensive, split model of treatment (where care is divided between a psychiatrist and a non-MD therapist) [28]. Despite the initial price difference, integrated care can be more cost-effective overall. Moreover, the time constraints that clinicians face in busy practices limit their ability to collaborate effectively. A recent study reveals that increased time pressure leads to fewer diagnoses being recorded during a visit and results in more scheduled and unscheduled follow-up care. Additionally, the study shows some evidence of an increase in low-value care, a reduction in preventive care, and a decline in opioid prescriptions [29]. Psychiatrists and psychotherapists typically operate on tight schedules, making it difficult for them to engage in meaningful discussions about patient care, and often resulting in fragmented treatment.

### 2.11. The Scarcity of Trained Mental Health Professionals Skilled in BD

The scarcity of trained mental health professionals skilled in BD-specific psychotherapies is another major barrier. Evidence suggests that specialized therapies like CBT and FFT, which are shown to be effective in treating BD, are not always accessible due to a shortage of trained therapists. The shortage of the healthcare workforce is global, and especially started peeking again during the COVID-19 pandemic in 2019. The current workforce cannot provide care to all those seeking it [30]. Furthermore, treatment adherence is often hampered by patients’ perceptions that psychotherapy is unnecessary when they feel stable, or by challenges in finding therapists with experience in BD. A recent study identifies nine drugs and several non-pharmacological approaches being developed to address factors causing treatment resistance in schizophrenia, major depressive disorder, bipolar affective disorder, and obsessive compulsive disorder (OCD). Key challenges contributing to treatment resistance include the heterogeneity of conditions, the absence of consensus criteria, the limited understanding of neurobiology, under-investment in research, and a lack of effective treatments [31]. Addressing these issues requires systematic changes, including better communication between care providers, increased access to specialized training for clinicians, and efforts to reduce stigma surrounding mental healthcare. Furthermore, healthcare systems need to prioritize providing integrated care, improving the coordination of services across providers, and ensuring that patients have access to both psychotherapy and pharmacotherapy when necessary.

In addition, recent research has emphasized the role of digital tools in overcoming these barriers. Technology-assisted interventions, such as online or mobile apps for mood tracking, have shown promise in increasing engagement and providing continuous support for BD patients [32]. These tools can help bridge the gap between pharmacological and psychotherapeutic treatments, providing a more accessible and convenient option for patients, especially those in remote or underserved areas.

Addressing these obstacles requires ongoing efforts to improve collaboration, training, and access to resources. A truly integrated approach will not only involve pharmacological and psychotherapeutic treatments but also a holistic consideration of the challenges BD patients face in adhering to their treatment plans.

### 2.12. Future Therapy Challenges: Considering Costs, Therapist Availability, and Patient Adherence

While this manuscript primarily focuses on the benefits of diverse psychotherapeutic interventions for bipolar disorder (BD), it is crucial to acknowledge the challenges patients face in accessing these treatments. Cost, therapist availability, and patient adherence are significant barriers that often hinder the effectiveness and reach of psychotherapy. Many individuals with BD struggle to find affordable therapy options or face long waiting times for specialized therapists, especially in rural or underserved areas. Additionally, some patients may experience difficulties adhering to treatment due to factors such as financial constraints, lack of social support, or the time commitment required for regular sessions.

Given the multifaceted nature of BD and the need for tailored therapeutic approaches, it is essential to consider how these obstacles can be mitigated to improve treatment outcomes. One potential solution lies in leveraging digital health technologies, which can provide more accessible and cost-effective options for patients. Teletherapy and mobile applications have the potential to reduce the burden of in-person therapy by offering virtual sessions and providing tools for self-monitoring, mood tracking, and therapeutic interventions. These digital solutions can be particularly useful in addressing adherence issues, as they allow patients to engage in therapy at their own convenience and from the comfort of their homes.

In the following subsections, we will explore telemedicine and mobile application options for BD treatment in greater detail, examining their potential to overcome these barriers and enhance treatment access.

### 2.13. Top-Notch Research Designs for Future Bipolar Disorder Treatment Approaches: A Systematic Review and Meta-Analysis

Emphasizing the role of meta-analyses in advancing research, future studies should prioritize conducting a comprehensive meta-analysis comparing the efficacy of various psychotherapeutic interventions for bipolar disorder. Such analyses would synthesize data from multiple studies, providing robust statistical insights into the relative effectiveness of therapies like CBT, IPSRT, and mindfulness-based approaches. By aggregating empirical data, meta-analyses can offer clearer conclusions about treatment outcomes, informing clinical practice. Additionally, further research should focus on RCTs to refine treatment protocols and explore the integration of digital tools alongside psychotherapies for more personalized, accessible care.

### 2.14. Integrating Digital Tools with Traditional Therapeutic Methods, Such as Psychotherapy

In light of the evolving landscape of mental health treatment, we recognize the promising potential of integrating digital tools with traditional therapeutic methods, such as psychotherapy. Digital interventions, including mobile apps, online therapy platforms, and virtual reality, have been shown to enhance therapeutic outcomes by providing scalable and accessible solutions. When combined with conventional psychotherapy, these digital tools offer opportunities for personalized, real-time support, and data-driven treatment adjustments.

For instance, digital tools can facilitate continuous monitoring of a patient’s progress, providing therapists with real-time feedback and allowing for more tailored therapeutic interventions. Additionally, the use of digital tools may help address barriers such as accessibility, cost, and the stigma associated with traditional psychotherapy, making mental healthcare more inclusive and widespread.

Future research could focus on exploring how these digital and psychotherapeutic interventions can be effectively integrated, with a particular focus on their synergistic effects. By studying these innovative combinations, we can deepen our understanding of their mechanisms and optimize their implementation to improve patient outcomes.

Several mobile applications have been developed to assist individuals with BD in preventing relapses, managing circadian rhythms, and aiding in diagnostics.

A systematic review of mobile apps for BD reveals an increasing reliance on smartphones for information dissemination and disease management [33]. However, this study highlights significant gaps in the current app offerings, suggesting that many apps are developed without considering research data or adhering to best clinical practice guidelines. These findings emphasize the need for caution when clinicians recommend apps to complement treatment, as the quality of available apps may vary widely. Furthermore, policymakers and the research community must address ways to ensure app quality, as current app development does not always align with the latest scientific evidence. There is a clear opportunity for mental health research to focus on creating evidence-based, high-quality mobile interventions for BD management. Given the rapid evolution of both research and technology, it is essential to establish new frameworks that can bridge the gap between these domains, ensuring that effective, evidence-based apps are available to users. Future research should prioritize the development of mobile interventions grounded in clinical evidence, focusing on areas such as relapse prevention, mood tracking, and integrating digital tools with psychotherapeutic approaches.

## 3. Conclusions

**Figure A1:**
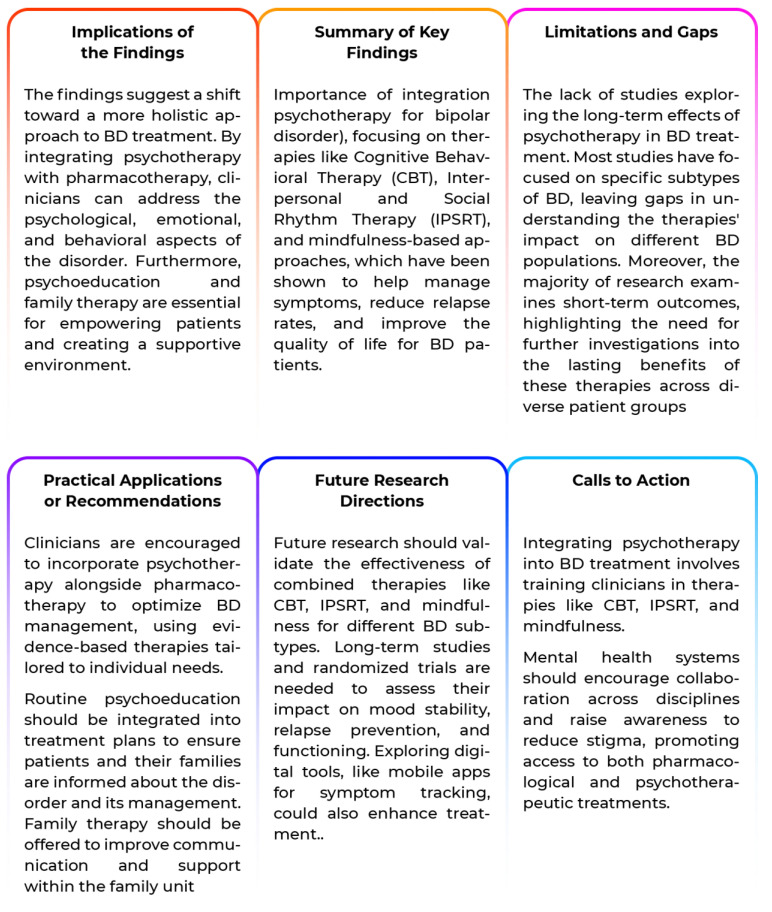
What now? Implications of findings, summary of key findings, limitations and gaps, practical applications or recommendations, future research directions, and calls to action.

Psychotherapy plays a vital role in the comprehensive treatment of BD. Evidence-based approaches such as CBT, IPSRT, and mindfulness-based therapies offer robust tools for managing mood episodes, enhancing coping strategies, and preventing relapse. A multifaceted treatment model that integrates pharmacological treatments with psychotherapy offers the best potential for improving patient outcomes. By incorporating psychoeducation and family therapy, clinicians can foster a supportive environment for individuals with BD. This integrated approach aligns with a more holistic and patient-centered model of care, addressing the multifactorial nature of BD. Moving forward, further research is needed to refine and optimize psychotherapeutic interventions, with a focus on individualized treatment plans tailored to the needs of each patient. In this way, psychotherapy can significantly contribute to long-term management and improved quality of life for those living with bipolar disorder. We provide Figure A1. With What now? Implications of findings, summary of key findings, limitations and gaps, practical applications or recommendations, future research directions, and calls to action.

## Data Availability

No new data were created or analyzed in this study. Data sharing is Data are contained within the article.

## Abbreviations

The following abbreviations are used in this manuscript:

BD	Bipolar disorder
BD-1	Bipolar disorder Type I
BD-2	Bipolar disorder Type II
BRIEF	Behavior Rating Inventory of Executive Function
CBT	Cognitive Behavioral Therapy
DSM-5	Diagnostic and Statistical Manual of Mental Disorders 5
FFT	Family-Focused Therapy
FrSBe	Frontal Systems Behavior Scale
IPSRT	Interpersonal and Social Rhythm Therapy
MBCT	Mindfulness-Based Cognitive Therapy
OCD	Obsessive compulsive disorder
RCTs	Randomized controlled trials

## Appendix A

**Table A1:** Effects of psychotherapy on bipolar disorder symptoms and cognitive, emotional, and behavioral pathways.

Symptoms of Bipolar Disorder	Type of Psychotherapy	Cognitive Pathways Affected	Emotional Pathways Affected	Behavioral Changes Targeted	Outcome per Symptom Anticipated
Depression	Cognitive Behavioral Therapy (CBT)	Negative thought patterns, cognitive distortions	Decreased negative emotional reactivity	Improved problem-solving, adaptive coping strategies	Reduced depressive symptoms through restructuring negative thought patterns and emotional regulation
	Interpersonal and Social Rhythm Therapy (IPSRT)	Disruption of sleep–wake cycles	Reduced sadness and hopelessness	Regular sleep routines, improved inter- personal relatios	Stabilized mood with consistent daily rhythms, reducing depressive episodes
	Mindfulness-Based Cognitive Therapy (MBCT)	Rumination, negative thought patterns	Increased insight of depressive triggers	Reduced automatic emotional reactions	Decreased relapse of depression through mindfulness and present-moment awareness
	Family-Focused Therapy (FFT)	Dysfunctional family dynamics	Reduced familial stress and anxiety	Improved communication skills, strengthened family support	Reduced depressive symptoms by increasing family support and understanding
Mania	Cognitive Behavioral Therapy (CBT)	Distorted thinking, impulsive decision- making	Increased emotional regulation	Impulse control, coping with high-energy states	Reduced manic episodes by improving self-awareness and controlling impulsivity
	Interpersonal and Social Rhythm Therapy (IPSRT)	Disrupted social rhythms	Increased irritability	Regular sleep–wake cycles, sable social routines	Decreased manic episodes through stabilization of daily routines and improved socialization
	Interpersonal and Social Rhythm Therapy (IPSRT)	Unstable sleep–wake cycles	Emotional dys- regulation	Stabilized daily routines, improved communication	Reduced frequency and intensity of mood swings through routines and social support

**Table A2:** Early signs of relapse in bipolar disorder, with corresponding potential interventions for family members/caregivers, therapists, and the patient themselves.

Early Signs of Relapse	Interventions (Family Members/Caregivers)	Interventions (Therapist)	Interventions (Patient)
Increased mood swings (high or low)	Observe and provide emotional support. Encourage routine and consistency.	Monitor mood patterns and adjust treatment plan.	Track mood changes in a journal. Practice mindfulness techniques.
Sleep disturbances (insomnia or hypersomnia)	Encourage healthy sleep hygiene and a consistent daily routine.	Assess sleep patterns, consider adjusting medications if needed.	Maintain a regular sleep schedule. Limit caffeine intake and exercise.
Impulsivity or risky behavior	Set clear boundaries. Communicate concerns and ensure patient safety.	Work on impulse control strategies and coping mechanisms.	Practice mindfulness. Utilize coping skills to manage impulses.
Social withdrawal or isolation	Encourage regular social interaction, offer companionship, check in frequently.	Explore underlying causes. Offer CBT or social skills training.	Stay connected with family/friends; engage in support groups.
Unusual thoughts or speech patterns	Stay calm and nonjudgmental. Encourage seeking professional help.	Monitor symptoms closely, adjust therapy techniques if necessary.	Be aware of signs and notify the therapist if symptoms worsen.
Increased energy or restlessness	Help manage overactivity. Suggest calming activities or relaxation techniques.	Assess if symptoms are indicative of mania and adjust medications.	Practice relaxation exercises; avoid over-exertion.
Difficulty concentrating	Be patient. Offer reminders and structure for daily tasks.	Use cognitive techniques to improve focus and task management.	Break tasks into smaller steps; stay organized and prioritize.
Feelings of hopelessness or worthlessness	Provide emotional support and reassurance. Encourage seeking professional help.	Engage in Cognitive Behavioral Therapy (CBT) to address negative thoughts.	Practice self-compassion. Challenge negative thinking patterns.
